# Nursing Affairs in Ireland

**Published:** 1917-10-27

**Authors:** 


### NURSING AFFAIRS IN IRELAND.

### District Nurses.

JUST now in various parts of Ireland well-disposed voluntary workers, including the clergy of all denominations, are engaged in raising money by the usual popular artifices for the upkeep of the district nurse. Concerts, bazaars, house-to-house collections, and personal appeals are the commonest means, and the advertisements generally point out that if the response is not adequate the nurse's services will have to be dispensed with. Voluntary effort is, of course, the natural forerunner of State or Municipal reform, and therefore probably in the fulness of time the district nurse may hope to become a recognised factor in the life of the community. Nevertheless, this kind of appeal must be repugnant to the feelings of the trained nurse. The announcement of a bazaar or a *fête* in a shopkeeper's window, accompanied by the invitation, "Buy a ticket for the support of the district nurse," is offensive, however well meant. Such a nurse's position, moreover, is obviously precarious, and it is natural that a more attractive and more permanent appointment should be sought. Now, it must be observed that the voluntary workers, who are doing excellent propaganda work in the cause of child welfare and public health generally, are not concerned with the welfare of the nurse; or, if they are, their interest is altogether subordinate to the main object which they have in view. The Infants' Welfare Act of 1917 (?) provides for municipalities using money on hand, or striking a small rate, for the purposes of the Act, which, if adopted, would entitle the Councils to claim half of the expense of the nurses' upkeep from the Local Government Board. But, as it happens, very few Councils have so far had the public spirit to take advantage of this opportunity, and in the meantime the one person on whose skill and devotion to duty the whole of the child-welfare problem depends occupies the unenviable position of being advertised as an object of public charity.

I have more than once pointed out that the economics of nursing are utterly neglected in Ireland, and, vital though State Registration may be to the profession, it cannot, save in a small way, help in the direction of material benefit for the rank and file. With more or less obscurity of vision nurses realise that none of the existing nursing institutions are concerned with the economic side—the questions of status, pay, hours of duty, pension, and so forth—and hence the almost complete lack of enthusiasm as to membership. The welfare of the trained nurse is a matter of public interest. It is of paramount importance to the community that the health of the poor and the preservation of infants from the ravages of disease should be looked after, and unless the supply of well-trained and efficient nurses is adequate this is clearly impossible. Surely this aspect of nursing should be regarded as of prime importance by the College of Nursing. If it were, I venture to suggest that there would be no lack of membership. Enrolment on a State Register is something, but it ought to be by no means the goal of achievement. Indeed, if by some misfortune it were to be indefinitely postponed, it is obvious that many other reforms of immense practical value might be accomplished independently. Personally, I have little doubt that if the public were appealed to there would be a ready response. Hundreds of excellent women workers of position and influence in Ireland are now engaged in endeavouring to solve some of the problems of the poor. The nurse, being an essential factor to most of these problems, ought to be brought within the ambit of their operations.

### "Our Day" Pageant.

This was an unqualified success in Dublin. Some timorous people feared a Sinn Fein opposition, but Sinn Fein recognised the neutrality of the Red Cross, and it was with delight that Dublin citizens witnessed the crowds of poor women, who thronged round the procession as it passed, shouting "God bless the nurses!" One feature of the pageant was an exhibition of an operating-table and the usual theatre equipment, with doctors and nurses standing clad as for an operation. A sum of almost £20 was thrown in small coins on to this particular motor-lorry as it passed along the streets. Dublin was gay with bunting, and "Our Day" in the city was an unqualified success.

